# The spectrum of phenylalanine hydroxylase variants and genotype–phenotype correlation in phenylketonuria patients in Gansu, China

**DOI:** 10.1186/s40246-023-00475-7

**Published:** 2023-04-25

**Authors:** Chuan Zhang, Pei Zhang, Yousheng Yan, Bingbo Zhou, Yupei Wang, Xinyuan Tian, Shengju Hao, Panpan Ma, Lei Zheng, Qinghua Zhang, Ling Hui, Yan Wang, Zongfu Cao, Xu Ma

**Affiliations:** 1 Gansu Province Medical Genetics Center,Gansu Provincial Clinical Research Center for Birth Defects and Rare Diseases, Gansu Provincial Maternity and Child-Care Hospital, Lanzhou, China; 2 National Research Institute for Family Planning , National Human Genetic Resources Center, Beijing, China; 3 Department of Nosocomial Infection Management, Lanzhou University Second Hospital, Lanzhou, China; 4 Prenatal Diagnostic Center, Beijing Obstetrics and Gynecology Hospital, Capital Medical University, Beijing, China

**Keywords:** PKU, *PAH*, Variant spectrumt, Genotype–phenotype correlation, Neonatal genetic screening

## Abstract

**Background:**

Phenylketonuria (PKU) is a common, congenital, autosomal recessive, metabolic disorder caused by Phenylalanine hydroxylase (*PAH*) variants.

**Methods:**

967 PKU patients from Gansu, China were genotyped by Sanger sequencing, multiplex ligation-dependent probe amplification, and whole exome sequencing. We analyzed the variants of *PAH* exons, their flanking sequences, and introns.

**Results:**

The detection of deep intronic variants in *PAH* gene can significantly improve the genetic diagnostic rate of PKU. The distribution of *PAH* variants among PKU subtypes may be related to the unique genetic background in Gansu, China.

**Conclusion:**

The identification of *PAH* hotspot variants will aid the development of large-scale neonatal genetic screening for PKU. The five new *PAH* variants found in this study further expand the spectrum of *PAH* variants. Genotype–phenotype correlation analysis may help predict the prognosis of PKU patients and enable precise treatment regimens to be developed.

## Introduction

Phenylketonuria (PKU, OMIM:261600) is the most common congenital, autosomal recessive, metabolism disorder and is caused by homozygous or compound heterozygous mutations in the Phenylalanine hydroxylase (*PAH*) gene on chromosome 12q23 [1]. The prevalence of PKU varies significantly among ethnicities and geographic regions of the world [1]. The incidence of PKU is approximately 1:10,000 live births in the USA [2]. In Europe, the incidence of PKU in Finland is relatively low at 1:112,000 live births [3]; however, in the Karachay-Cherkess Republic of Russia, incidence ranges from 1:850 in newborns to 1:332 among Karachay nationals [4]. In Asia, the incidence of PKU in Japan and Thailand is relatively low [5, 6]. In China, the average incidence of PKU is 15,924 [7]; however, Gansu Province has the highest incidence of PKU in China at 1/3420 [8].

PKU can be classified into three subtypes according to the phenotype: mild hyperphenylalaninemia (MHP; blood phenylalanine (Phe) concentrations of 120–360 μmol/L; regular follow-up is recommended but no treatment required); mild phenylketonuria (mPKU; blood Phe concentrations of 60–1200 μmol/L), and classic phenylketonuria (cPKU; blood Phe concentrations of ≥ 1200 μmol/L). mPKU and cPKU patients require dietary therapy [9, 10]. Untreated PKU patients can develop global developmental delay or severe irreversible intellectual disability, as well as growth failure, hypopigmentation, motor deficits, ataxia, and seizures [11]. Low rates of PKU screening and treatment affect the quality of life of affected individuals and the infant mortality rate [12, 13]. Therefore, early screening, genetic diagnosis, and treatment are important for reducing the damage that PKU can cause in patients.

Studying the spectrum of *PAH* variants is helpful for screening high-risk groups and to provide prenatal diagnosis and prenatal genetic counselling. As of November 2, 2022, the PAHvdb database (http://www.biopku.org/home/pah.asp) had collated 1583 PAH variants, including missense, frameshift, synonymous, splicing, untranslated region, and large-scale deletion variants. The clinical application of whole genome sequencing technology has led to the discovery of increasing numbers of pathogenic deep intron variants (more than 100 bp from the exon–intron boundary). Pathogenicity analysis of deep intron *PAH* variants improves the diagnostic rate of PKU [14].

In this study, we performed exon and intron variant analysis of *PAH* in a large cohort of 967 PKU families from Gansu Province, Northwest China. The findings can inform follow-up treatment, genetic counseling, and prenatal genetic counselling for PKU patient families. Furthermore, studying the molecular genetics of *PAH* can also provide basic data for improving screening and diagnostic measures for PKU.

## Materials and methods

### DNA samples

According to clinical features and newborn screening, 967 cases of PKU were diagnosed in the Medical Genetics Center of Gansu Provincial Maternity and Child-Care Hospital between January 2012 and December 2021. Among these patients, 407 were cPKU, 403 were mPKU and 157 were MHP. This study was undertaken according to the tenets of the Declaration of Helsinki 1975 and its later amendments. The study protocol was approved by the Ethics Committee of the Gansu Provincial Maternity and Child-Care Hospital. Written informed consent was obtained from all study participants or their legal guardians.

### Genomic DNA preparation

Genomic DNA was extracted from peripheral blood samples (2–3 mL) of patients and their parents using the Tiangen DNA extraction kit (Tiangen Biotech, China). DNA quality and quantification were assessed using a NanoDrop 2000 (Thermo Fisher, USA).

### Sanger sequencing

Thirteen PCR primer pairs, targeting exons and the flanking sequences of *PAH* were designed using Primer Premier 5.0 software (Premier Biosoft International, Palo Alto, CA, US) [13]. PCR products were purified on 2% agarose gels then bi-directionally sequenced using the BigDye Terminator v3.1 Cycle Sequencing Kit (Applied Biosystems, USA) on an ABI 3500DX Genetic Analyzer (Applied Biosystems).

### Whole exome sequencing and bioinformatics analysis

Whole exome sequencing was performed using an Agilent SureSelect Human All Exon V6 Kit (Agilent Technologies Inc., USA) on an Illumina NovaSeq 6000 platform (Illumina Inc., CA, USA). Data analysis and variant curation were performed with Pgenomics software (https://www.pgenomics.cn/). Variants were described according to the nomenclature recommended by the Human Genome Variation Society (www.hgvs.org/). Variant frequencies were searched for in the GnomAD (http://gnomad.broadinstitute.org/), Exome Sequencing Project (ESP,http://evs.gs.washington.edu) and SNP (dbSNP) (http://www.ncbi.nlm.nih.gov/projects/snp) databases. Candidate variants were confirmed in the parents of each family by Sanger sequencing.

### Large-scale deletion/duplication analysis

The detection of large-scale deletion/duplication *PAH* variants was performed using the SALSA MLPA P055 PAH kit (MRC Holland, Netherlands) according to the manufacturer’s protocol. Copy number variations of *PAH* exons were analyzed using Coffalyser software (MRC Holland, Netherlands).

### Statistical analysis

SPSS 22.0 software(IBM Corp., America) was used for statistical analysis. The χ^2^ test was used to analyze distribution differences of each pathogenic mutation locus among different PKU subtypes. After Bonferroni correction, *p* < 0.05 was considered statistically different.

## Results

Among the 967 PKU patients in this study, 407 were cPKU, 403 were mPKU, and 157 were MHP patients. We detected, 1909 variants in 1934 alleles, with a detection rate of 98.70%. The detection rate in cPKU families was 100%, in mPKU families 98.88%, and in MHP families 94.90%. The gene diagnosis rate was 89.81% for MHP, 98.01% for mPKU, 100% for cPKU, and 97.52% for the 967 PKU patients overall (Table 1).

**Table 1:** Gene diagnosis rate of PKU patients

Classification	Patient number	Number of genetic diagnoses	Proportion of genetic diagnoses (%)
MHP	157	141	89.81
mPKU	403	395	98.01
cPKU	407	407	100
Total	967	943	97.52

Two allelic gene variants were detected in 943 of the 967 PKU patients, only one heterozygous variant was detected in 23 patients, and definite or suspected *PAH* gene variants were not detected in one patient. Parents source verification was performed for all the variants detected in the PKU patients. The variants in 943 patients were all derived from parents. Among the 943 PKU patients with definite genetic diagnosis, 879 were caused by complex heterozygous variants and 64 were caused by homozygous variants.

The 1909 allele variants detected in 967 patients consisted of 185 different variants. Among these, the most frequent was c.728G > A, followed by c.611A > G, c.721C > T, c.1068C > A, and c.442-1G > A. The cumulative frequency of the first 22 hotspot mutations reached 70.9%. Of the 182 variants, c.160_163delTTAT, c.305T > A, c.376C > T, c.599C > T, c.694_696delCAGinsTAA were novel and not reported in ClinVar or HGMD databases. According to the American College of Medical Genetics and Genomics (ACMG) guidelines, c.160_163delTTAT, c.376C > T, c.694_696delCAGinsTAA were categorized as pathogenic, the evidences of them were PVS1, PM2, PM3, c.305T > A and c.599C > T were likely pathogenic, and the the evidences were PM1, PM2, PM3, PM5 (Table 2).

**Table 2:** Pathogenicity analysis of novel variants of *PAH* (NM_000277)

c.DNA change	p.change	Type	Classification	Evidences
c.160_163delTTAT	L54fs*6	Frameshift	Pathogenic	PVS1,PM2,PM3
c.305T > A	I102N	Missense	Likely pathogenic	PM1,PM2,PM3 PM5
c.376C > T	Q126*	Nonsense	Pathogenic	PVS1,PM2,PM3
c.599C > T	T200I	Missense	Likely pathogenic	PM1,PM2,PM3,PP3
c.694_696delCAGinsTAA	Q232*	Nonsense	Pathogenic	PVS1,PM2,PM3

Among the 185 variants detected in PKU patients, 120 were missense variants, accounting for 64.86%, 28 were splicing variants, accounting for 15.14%, and 19 were nonsense variants, accounting for 10.27% (Table 3). At the exon/intron level, the largest number of variants (27) was detected in exon 7, followed by exon 11 and then exon 12 (Table 4). Among the 1909 detected alleles, exon 7 had the largest number of alleles with 570, followed by exon 11 and then exon 12 (Table 4). Although only two variants were detected in intron 4, the number of alleles was high at 83. When Sanger sequencing was used to detect *PAH* variation, 71.9% of the mutation sites could be detected by amplification sequencing of exons 6, 7, 11, and 12 and their flanks (Table 5).

**Table 3:** Distribution of *PAH* gene variation types

Type	Number	Frequency (%)
Missense	120	64.86
Splice	28	15.14
Nonsense	19	10.27
Frameshift	6	3.24
Large-scale deletion	9	4.86
Large-scale duplication	2	1.08
5'UTR	1	0.54
Total	185	100

**Table 4:** Analysis of *PAH* gene variation distribution

Position	Variant type		Allele of variation	
	Number	Frequency	Number	Frequency
Exon 1	1	0.54%	1	0.05%
Intron 1	0	0.00%	0	0.00%
Exon 2	5	2.70%	61	3.20%
Intron 2	3	1.62%	15	0.79%
Exon 3	12	6.49%	175	9.17%
Intron 3	0	0.00%	0	0.00%
Exon 4	3	1.62%	5	0.26%
Intron 4	2	1.08%	83	4.35%
Exon 5	15	8.11%	71	3.72%
Intron 5	0	0.00%	0	0.00%
Exon 6	17	9.19%	182	9.53%
Intron 6	2	1.08%	3	0.16%
Exon 7	27	14.59%	570	29.86%
Intron 7	6	3.24%	69	3.61%
Exon 8	9	4.86%	25	1.31%
Intron 8	2	1.08%	2	0.10%
Exon 9	8	4.32%	21	1.10%
Intron 9	1	0.54%	1	0.05%
Exon 10	11	5.95%	44	2.30%
Intron 10	2	1.08%	8	0.42%
Exon 11	19	10.27%	250	13.10%
Intron 11	4	2.16%	24	1.26%
Exon 12	18	9.73%	188	9.85%
Intron 12	5	2.70%	38	1.99%
Exon13	1	0.54%	1	0.05%
Large-scale deletion	9	4.86%	69	3.61%
Large-scale duplication	2	1.08%	2	0.10%
5'UTR	1	0.54%	1	0.05%
Total	185	100.00	1909	100.00

**Table 5:** Number of alleles covered by *PAH* gene exons and their flanking sequences

Position	Allele number	Frequency of (%)	Frequency of accumulation (%)
Exon 7	639	35.21	35.21
Exon 11	256	14.10	49.31
Exon 12	226	12.45	61.76
Exon 6	184	10.14	71.90
Exon 3	175	9.64	81.54
Exon 4	88	4.85	86.39
Exon 2	76	4.19	90.58
Exon 5	71	3.91	94.49
Exon 10	49	2.70	97.19
Exon 8	27	1.49	98.68
Exon 9	22	1.21	99.89
Exon 1	1	0.06	99.94
Exon13	1	0.06	100.00

There were 40 *PAH* variations with allele frequency greater than or equal to 10. The distribution difference of the following 23 loci among the three PKU subtypes reached statistical significance after Bonferroni correction: c.728G > A, c.611A > G, c.721C > T, c.1068C > A, c.442-1G > A, c.1238G > C, c.1197A > T, c.842 + 2 T > A, c.331C > T, c.158G > A, c.208_210delTCT, EXUp_1del, c.782G > A, c.1199G > A, c.1301C > A, c.764T > C, c.1256A > G, c.740G > T, c.526C > T, c.1252A > C, EX1-1del, c.1174T > A and c.472C > T. The varisnts c.1199G > A, c.1301C > A, c.764T > C, c.740G > T, c.526C > T, c.1222C > T, c.498C > G, c.168 + 5G > C, c.472C > T and c.722delG were only detected in mPKU and cPKU and not in MHP, indicating that these sites are more likely to cause mPKU and cPKU.

According to the frequency of 23 variation sites with statistically significant distribution differences among the three PKU subtypes (all *p* < 0.001), the PKU subtype with the highest or lowest site frequency was designated as the experimental group, and the other two groups as the control group. The distribution frequencies of c.728G > A, c.1068C > A, c.442-1G > A, c.331C > T, and c.764T > C in the cPKU group were higher than in the MHP + mPKU group (*p* < 0.005, Table 6). The distribution frequencies of c.721C > T, c.208_210delTCT, c.1301C > A, and c.1252A > C in the mPKU group were higher than in the MHP + cPKU group (*p* < 0.007, Table 6). The distribution frequencies of c.158G > A, c.1256A > G, and c.1174T > A in MHP were higher than in the mPKU + cPKU group (all *p* < 0.008, Table 6).

**Table 6:** Comparison of distribution difference between *PAH* genotype and PKU subtype

Variant	cPKU (n = 407)	MHP + mPKU (n = 560)	χ^2^	*p*1
	Frequency	Frequency		
c.728G > A	148 (36.36%)	144 (25.71%)	12.68	< 0.001
c.1068C > A	56 (13.76%)	35 (6.25%)	15.59	< 0.001
c.611A > G	50 (12.29%)	44 (7.86%)	5.27	0.022
c.442-1G > A	46 (11.30%)	31 (5.54%)	10.69	0.001
c.1238G > C	37 (9.09%)	35 (6.25%)	2.76	0.097
c.331C > T	41 (10.07%)	21 (3.75%)	15.71	< 0.001
c.764T > C	19 (4.67%)	8 (1.43%)	9.11	0.003
c.526C > T	12 (2.95%)	8 (1.43%)	2.69	0.101
EX1-1del	9 (2.21%)	2 (0.36%)	7.21	0.007
c.472C > T	8 (1.97%)	2 (0.36%)	5.96	0.015

Variant	mPKU (n = 403)	MHP + cPKU (n = 564)	χ^2^	*p*2
	Frequency	Frequency		
c.721C > T	66 (42.04%)	25 (4.43%)	37.81	< 0.001
c.842 + 2T > A	33 (8.19%)	31 (5.50%)	2.76	0.097
c.208_210delTCT	26 (6.45%)	16 (2.84%)	7.39	0.006
c.1301C > A	22 (5.46%)	6 (1.06%)	16.15	< 0.001
c.1252A > C	17 (4.22%)	2 (0.35%)	18.22	< 0.001
c.1199G > A	17 (4.22%)	17 (3.01%)	0.18	0.675
c.740G > T	11 (2.73%)	11 (1.95%)	0.64	0.423

Variant	MHP (n = 157)	mPKU + cPKU (n = 810)	χ^2^	*p*3
	Frequency	Frequency		
c.158G > A	51 (32.48%)	4 (0.49%)	250.89	< 0.001
c.1256A > G	15 (9.55%)	7 (0.86%)	44.67	< 0.001
c.1174T > A	9 (5.73%)	1 (0.12%)	40.43	< 0.001
c.1197A > T	4 (2.55%)	64 (7.90%)	5.77	0.016
EXUp_1del	4 (2.55%)	36 (4.44%)	1.19	0.275
c.782G > A	2 (1.27%)	35 (4.32%)	3.32	0.069

Bonferroni correction, *p*1 < 0.005 was statistically significant; *p*2 < 0.007 was considered statistically significant. *p*3 < 0.008 was considered statistically significant

Additionally, we found 64 patients with PKU caused by homozygous variants, with 23 variants detected, among which two were detected in MHP, 10 in mPKU and 14 in cPKU (Table 7). There were only two cases of MHP caused by homozygous variants, which were c.721C > T/c.721C > T and c.158G > A/c.158G > A (Table 7). c.728G > A was the most frequent homozygous mutation, in 13 mPKU and 17 cPKU patients (Table 7). The c.721C > T/c.721C > T homozygous mutation caused mPKU in two cases, but no patients with homozygous mutations at this site were found in cPKU (Table 7). cPKU was also caused by large homozygous deletions, EX3del, EX1-2del and EX1-1del. In addition, homozygosity for c.442-1G > A and c.1238G > C was found in cPKU patients, but not in MHP or mPKU patients (Table 7).

**Table 7:** Distribution of variation sites in PKU patients with homozygous variation

PKU classification	Variant	Patient number
MHP	c.721C > T	1
	c.158G > A	1
mPKU	c.842 + 2T > A	2
	c.728G > A	13
	c.721C > T	2
	c.611A > G	1
	c.498C > G	1
	c.442-1G > A	2
	c.208_c.210delTCT	2
	c.194T > A	2
	c.1289T > C	1
	c.1197A > T	2
cPKU	EX3del	1
	EX1-2del	1
	EX1-1del	1
	c.782G > A	1
	c.764T > C	1
	c.740G > T	1
	c.728G > A	17
	c.665A > G	1
	c.442-1G > A	3
	c.331C > T	1
	c.1238G > C	2
	c.1222C > T	1
	c.1199G > C	1
	c.1197A > T	2

## Discussion

Hyperphenylalaninemia is the most common hereditary disorder of amino acid metabolism worldwide. PKU is the main type of hyperphenylalaninemia and is an autosomal recessive genetic disease caused by mutation of the *PAH* gene, which encodes phenylalanine hydroxylase [15]. There are regional differences in the distribution of the three PKU subtypes. cPKU accounts for the highest proportion of PKU in Estonia at 93.5%, followed by 72.6% in Slovakia, while the average proportion of cPKU in Chinese PKU is 62.1% [1]. In this study, cPKU accounted for 42.09% of PKU patients, which was significantly lower than the average proportion in China. This may be related to the unique genetic background of the population in Gansu Province.

Variations in *PAH* are widely distributed in exons, introns, and upstream and downstream untranslated regions of the gene. At the time of writing, the PAHvdb database included 1583 variants. By analyzing the exons and introns of *PAH*, we identified 185 variants in 967 PKU patients. Among these the most common were missense variations, followed by splicing variations and nonsense variations. These were distributed throughout the gene, except in introns 1, 3 and 5. Exon 7 had the most alleles, followed by exons 11, 12, 6, and 3, intron 4 and exon 5, indicating that the above regions were the hotspots of *PAH* variation. However, other studies have reported European PKU patients to have *PAH* hotspots in exon 12 and intron 11 [16], indicating that the distribution of *PAH* variation sites varies with ethnicity.

Among the 185 *PAH* variations detected in this study, the highest allele frequency was for c.728G > A (15.40%), followed by c.611A > G (5.08%), c.721C > T (4.87%), c.1068C > A (4.77%), c.442-1G > A (4.19%). c.1238G > C (4.03%), c.1197A > T (3.61%), c.842 + 2T > A (3.35%), c.331C > T (3.25%) and c.158G > A (2.88%). After genotyping 796 Chinese PKU patients, Li et al. [17] found that the high allele frequency sites for *PAH* were c.728G > A (17.53%), c.611A > G (7.66%), c.1197A > T (5.84%), c.721C > T (5.4%), c.331C > T (4.77%), c.1068C > A (4.46%), c.1238G > C (4.33%), and c.442-1 G > A (3.77%). In another study of 808 Chinese PKU patients by Wang et al. [18], the high frequency *PAH* alleles were c.728G > A (17%), p.Y204_T236delinsS (7.4%), c.721C > T(7.2%), p.W356Efs*22 (5.4%), c.331C > T (4.4%), c.1068C > A (3.9%), c.1238G > C (3.8%) and c.442-1G > A (3.7%). The allele frequency of c.842 + 2T > A was relatively high and that of c.331C > T was relatively low in Gansu province. This indicates that there are regional differences in the frequency of *PAH* alleles in different regions of China.

There are significant differences in the detection rate and diagnostic rate of gene variants in patients with the three different types of PKU. The diagnostic rate of mPKU and cPKU is significantly higher than that of MHP [19, 20]. Among the 967 PKU patients in this study, the detection rate of the pathogenic allele was 98.70%, and the diagnostic rates of MHP, mPKU and cPKU were 89.81%, 98.01% and 100%, respectively. In this study, the detection rate of alleles in PKU patients and the gene diagnosis rate of the three PKU subtypes were higher than those in previous studies [18–26]. This is because we analyzed the deep introns of *PAH*. By detecting deep intron mutations in *PAH*, we confirmed the genetic diagnosis of 22 cases of PKU, further improving the genetic diagnosis rate of PKU patients; however, there were still 4.8% of PKU patients who did not receive a definite genotype diagnosis. This could be because some non-*PAH* genes, for example, *DNAJC12* may affect PAH function to increase blood Phe concentrations [27], or because of epigenetic factors [28]. Studies have shown that Long non-coding RNAs Pair and human HULC were associated with PAH and modulated enzymatic activities by facilitating PAH substrate and PAH-cofactor interactions [29, 30].

A large number of *PAH* variants retain varying degrees of PAH activity, and the differences in these enzyme activities correspond to different PKU phenotypes [31, 32]. Therefore, studying the spectrum of pathogenic PAH variation in PKU is essential for clarifying the association between PKU genotype and clinical phenotype and for informing PKU treatment, fertility guidance, genetic counseling, and prenatal diagnosis [1]. In addition, predicting the phenotype of PKU from the *PAH* genotype may have very important clinical value, especially in the treatment of patients with critical blood Phe concentration and for genetic counseling of patients’ families [1].

We found that the distribution frequency of *PAH* loci, c.728G > A, c.1068C > A, c.442-1G > A, c.331C > T, and c.764T > C in the cPKU group was higher than in the MHP + mPKU group (all *p* < 0.005, Table 6), indicating that these loci were the main pathogenic variants of cPKU. The distribution frequency of c.721C > T, c.208_210delTCT, c.1301C > A, and c.1252a > C in the mPKU group was higher than in the MHP + cPKU group (all *p* < 0.007, Table 6), and these loci were the main pathogenic variants of mPKU. The distribution frequency of c.158G > A, c.1256A > G, and c.1174T > A in MHP was higher than in the mPKU + cPKU group (all *p* < 0.008, Table 6), and these loci were the main pathogenic variants in MHP. c.1199G > A, c.1301C > A, c.764T > C, c.740G > T, c.526C > T, c.1222C > T, c.498C > G, c.168 + 5G > C, c.472C > T and c.722delG were the main pathogenic variants of mPKU and cPKU and were not detected in MHP (Table 6).

One case of MHP and two cases of mPKU (but no cPKU cases) were caused by c.721C > T/c.721C > T homozygosity. The blood Phe of these two mPKU patients were 363 μmol/L and 366 μmol/L. Therefore, we speculated that c.721C > T was associated with MHP and light mPKU. Homozygosity of c.728G > A was found in 13 cases of mPKU and 17 cases of cPKU, which further indicated that the carrier rate of c.728G > A was high in this region and was related to mPKU and cPKU. cPKU was also caused by homozygous deletion of large fragments, including EX3del, EX1-2del and EX1-1del. Patients homozygous for c.442-1G > A or c.1238G > C also presented cPKU. Therefore, we speculate that these sites are also main pathogenic variation sites of cPKU.

Establishment of a database curating hotspot mutations of PKU genes will aid the establishment of rapid, efficient, and low-cost genotyping procedures. These can be used to rapidly assess PKU patients to enable timely prevention and intervention treatments. They can also be used for newborn screening and for the screening of carriers in the pre-pregnancy population. At present, many commercial reagents are available for screening deafness gene hotspot mutations [33–36]. However, there is no clinical detection kit for screening *PAH* hotspot mutations in PKU, which is not conducive to the early screening and diagnosis of PKU patients.

In this study, we found that more than 70% of PKU patients were detected by screening the first 22 *PAH* hotspot mutations. Therefore, large-scale newborn screening could be performed to identify more than 70% of PKU patients. PCR-Sanger sequencing was performed on exons 6, 7, 11 and 12 of *PAH*, and the detection rate of PKU mutation sites in the local Gansu region was more than 70%, which could be used for the preliminary diagnosis of PKU.

In summary, this study is the largest sample size of PKU genotype phenotype study in a single area of China, we performed deep intron variant detection of *PAH*, which can improve the gene diagnosis rate of PKU. The *PAH* hotspot variants have the potential to provide basic data for large-scale neonatal genetic screening. The five new *PAH* variants found in this study further expand the spectrum of *PAH* variation. The analysis of genotype–phenotype correlations will help predict the phenotype of PKU patients and the tailoring of individualized treatment.

## Data Availability

The datasets used and/or analyzed during the current study are available from the corresponding author on reasonable request. The five novel variants have been submitted to the PAHvdb (http://www.biopku.org/pah/). The respective accession IDs of PAHvdb is PAH1790 for c.694_696delCAGinsTAA, PAH1791 for c.599C > T, PAH1792 for c.376C > T, PAH1793 for c. c.305T > A, and PAH1621 for c.160_163delTTAT.
